# Androgen receptor signaling is required for androgen-sensitive human prostate cancer cell proliferation and survival

**DOI:** 10.1186/1475-2867-5-8

**Published:** 2005-04-06

**Authors:** Qing Yang, Kar-Ming Fung, Wanda V Day, Bradley P Kropp, Hsueh-Kung Lin

**Affiliations:** 1 Department of Urology, University of Oklahoma Health Sciences Center, Oklahoma City, OK, USA; 2 Department of Pathology, University of Oklahoma Health Sciences Center, Oklahoma City, OK, USA; 3 Department of Veterans Affairs Medical Center, Oklahoma City, OK, USA

**Keywords:** androgen receptor, RNA interference, prostate cancer

## Abstract

**Background:**

Androgens and androgen receptors (AR) regulate normal prostate development and growth. They also are involved in pathological development of prostatic diseases, including benign prostatic hyperplasia (BPH) and prostate cancer (PCa). Antiandrogen therapy for PCa, in conjunction with chemical or surgical castration, offers initial positive responses and leads to massive prostate cell death. However, cancer cells later appear as androgen-independent PCa. To investigate the role of AR in prostate cell proliferation and survival, we introduced a vector-based small interfering RNA (siRNA). This siRNA targeted 5'-untranslated region of AR mRNA for extended suppression of AR expression in androgen-sensitive human prostate LNCaP cells.

**Results:**

The siRNA design successfully suppressed endogenous AR expression, as revealed by western blotting and immunofluorescence staining in LNCaP cells. LNCaP cells did not proliferate in the absence of AR and underwent apoptosis, based on elevated phospho-Histone H2B expression and higher number of apoptotic body as compared to control cells.

**Conclusion:**

We demonstrated that AR is vital for prostate cell proliferation and survival in this androgen-sensitive prostate cell line. These results further strengthen the hypothesis that AR can be a therapeutic target for treating androgen-sensitive stages of PCa. Unlike antiandorgens, however, siRNA targeting AR provides a direct inactivation of AR function through the suppression of AR protein expression.

## Background

Androgens are critical for the development and growth of normal prostate. They also are responsible for the development of prostate diseases, including benign prostatic hyperplasia (BPH) and prostate cancer (PCa). Androgen receptors (AR) transduce androgen signals in prostate cells to regulate the physiological and pathological development of the gland [1]. It is classically characterized that after ligand binding [mainly 5α-dihydrotestosterone (5α-DHT)] the ligand-AR receptor complex with associated proteins translocates into the nucleus, binds to the consensus sequence of androgen response elements (AREs) [2], and regulates androgen responsive genes (ARGs) expressions [3]. Conditions that activate abnormal AR *trans*-activation through AR mutations [4,5], amplification of AR [6], or androgen-independent signaling pathways [7,8] can lead to or be a result of the development of prostatic diseases or androgen refractory PCa.

Clinically, androgen ablation therapy is used to reduce AR ligand production or to block AR-mediated signaling. Finasteride, a 5α-reductase type 2 inhibitor, has been used for treating patients with BPH. Finasteride slows the progression of BPH by suppressing 5α-DHT synthesis [9]. Androgen ablation therapy for PCa has been achieved using compounds called antiandrogens. Antiandrogens compete with 5α-DHT for AR binding and intend to block AR-mediated signaling. Antiandrogens are classified, according to their chemical structure, as either steroidal antiandrogens (ie, cyproterone acetate or medroxyprogesterone acetate) or non-steroidal anti-androgens [ie, nilutamide (Anandron™), flutamide (Eulexin™), and bicalutamide (Casodex™)] [10]. Unfortunately, essentially all of the patients who show initially favorable responses to the androgen blockade eventually become refractory to this treatment [11].

Abruption of AR signaling has been examined in experimental models to investigate AR-mediated prostate cell physiology and pathophysiology. An antisense oligonucleotide [12], a hammerhead robozyme [13], and microinjection of an AR neutralizing antibody [14] have been designed to suppress AR expression or block AR-mediated signaling in *in vitro *models. The advance of RNA interference (RNAi) technology provides a tool to suppress gene expression through post-translational gene silencing (PTGS) [15]. This technology utilizes duplex small interfering RNAs (siRNAs) of about 19–23 nucleotides to suppress the expression of the homologous gene [16,17]. Wright *et al*. reported a successful use of a synthetic double-stranded (ds) siRNA to suppress endogenous AR expression in LNCaP cells [18]. In this study, we established a vector-based siRNA plasmid construct targeting the 5'-untranslated region (5'-UTR) of the AR in the same prostate cell line. We successfully achieved AR silencing in LNCaP cells, and further demonstrated that these cells could neither proliferate nor survive in the absence of the AR. We concluded that AR signaling is required for both prostate cell proliferation and survival, at least in androgen-sensitive states. Specific blocking of AR expression and AR-mediated signaling is required for a successful androgen blockade in patients with prostate diseases. In addition, the vector-based AR silencing design provides a tool to study AR-associated genomic and non-genomic effects and to identify potential pathways that cross-talk with AR.

## Results and discussion

**Figure 1:**
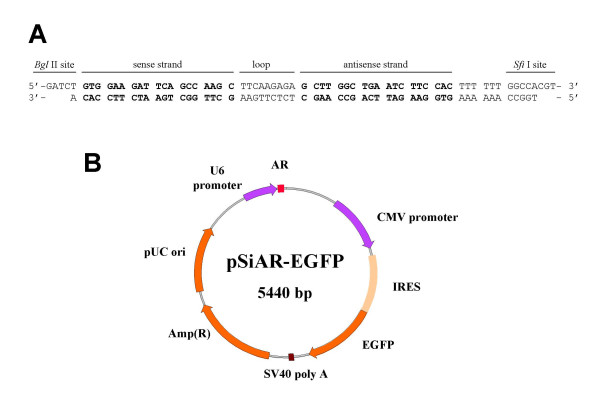
**A vector-based plasmid construct for suppression of AR expression in LNCaP cells. **(A) Two single-stranded oligonucleotides were synthesized consisting of a 19 nucleotide of -25 to -7 bp 5'-UTR of the AR mRNA separated by a short loop sequence from the reverse complement of the same sequence. The sense strand of synthesized oligonucleatides was ended with five thymidines as termination signal. The annealed ds DNA contained *Bgl *II and *Sfi *I restriction at its 5' and 3' end of the oligonucleotides, respectively. (B) Schematic drawing of the pSiAR-EGFP vector. The expression construct was designed to expression AR siRNA driven by U6 promoter for suppression of endogenous AR expression in mammalian cells. The expression construct also contained EGFP driven by the CMV promoter constitutive expression of GFP in target cells.

To identify the role of AR in AR-positive, androgen-sensitive LNCaP cells, we designed a vector-based siRNA plasmid construct to achieve "long-term" AR silencing. This construct contained the U6 promoter-driven sense and antisense strands of AR siRNA that have a termination signal consisting of 6 thymidines. This construct also included a constitutively expressed CMV promoter-driven green fluorescence protein (GFP), designated pSiAR-EGFP (Fig. 1). Because there is an adverse effect on androgen-sensitive prostate cell proliferation in the absence of the AR [12,14,18], the inclusion of the CMV promoter-driven GFP expression could be used to select and enrich AR-negative LNCaP cells following transfection. A construct, named pSiCon-EGFP, was established with a scrambled AR target sequence and served as a control. At 16 hours following transfection, GFP was expressed and detectible under a fluorescence microscope (data not shown).

### Plasmid siRNA construct targeting 5'-UTR of AR mRNA suppressed AR expression in LNCaP cells

**Figure 2:**
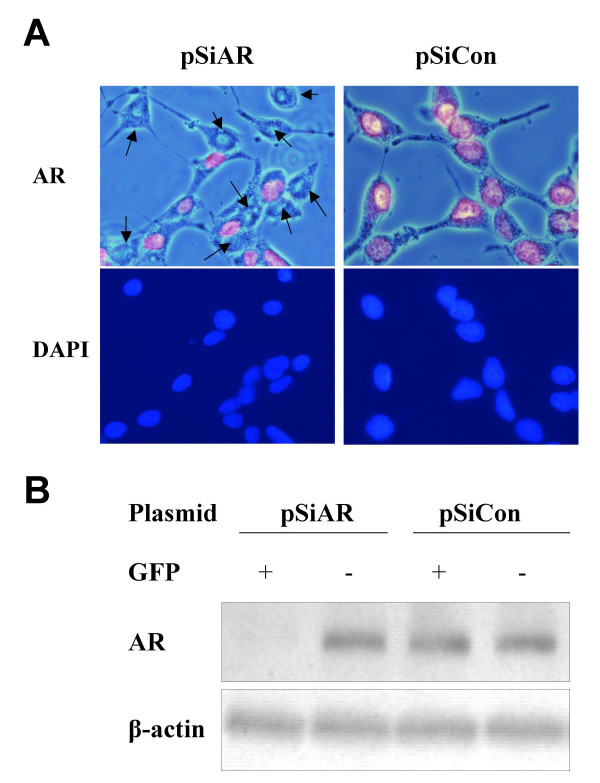
**Suppression of endogenous AR expression in LNCaP cells using the pSiAR-EGFP expression construct. **LNCaP cells were transfected with either the pSiAR-EGFP or the pSiAR-EGFP construct using the Lipofectemine 2000 protocol. (A) Immunocytochemical detection of AR expression in transfected cells. To determine AR expression in LNCaP cells, following transfection, cells were stained with mouse anti-human AR monoclonal antibody (1:100 dilution) followed by AlexaR FluorR 594 conjugated goat anti-mouse IgG secondary antibody (2 mg/ml) incubation. The AR staining was detected by fluorescent microscopy (BX51, Olympus). The images were composite between the red with the positive in AR staining with the phase-contract from the same field. Suppression of endogenous AR expression was demonstrated by the absence of red fluorescence staining as indicated by arrows. DAPI staining showed the number of cells in the same field. (B) Western blot analysis of AR expression in pSiAR-EGFP and control transfected cells. At 7 days following transfection, both GFP-positive and GFP-negative cells were collected through cell sorter; and cells were lysed to prepare cellular protein extracts. Aliquots of 20 μg total cellular protein were loaded into Tris-HCl gels and transferred to PDVF membranes. The AR expression was determined by incubating with mouse anti-human AR monoclonal antibody (1:500) followed by the HRP-conjugated anti-mouse IgG (1:125,000) secondary antibody incubation. Immunoreactive signals were detected using ECL. Levels of β-actin expression was also determined in each sample and used as protein loading control.

To determine whether the pSiAR-EGFP construct was capable of expressing siRNA, thereby suppressing AR expression in LNCaP cells, cells were transfected with pSiAR-EGFP or with pSiCon-EGFP construct. AR expression was monitored by staining these cells with mouse-anti-human AR monoclonal antibody. Immunocytochemical staining of LNCaP cells showed that portions of cells transfected with pSiAR-EGFP plasmid had no detectible AR expression, whereas cells transfected with pSiCon-EGFP continued to show AR expression in all cells (Figure 2A). However, at 2 weeks following transfection, there was no detectible GFP-positive or AR-negative LNCaP cells in the pSiAR-EGFP transfected group (data not shown). This suggested that either the cells lost their transfected pSilencer plasmids, or that the cells receiving the pSiAR-EGFP could, in fact, not survive in the absence of the AR.

To demonstrate that GFP can be used as a marker for selecting AR-negative LNCaP cells, pSiAR-EGFP or pSiCon-EGFP transfected cells were trypsinized 24 hours following transfection. GFP-positive cells were collected through a cell sorter to determine AR protein expression. Western blot analysis showed no detectible AR protein expression in GFP-positive cells transfected with pSiAR-EGFP. However, GFP-positive cells receiving the control pSiCon-EGFP construct continued to express AR protein (Figure 2B). This result demonstrated that GFP positive cells are AR negative cells.

The use of 5'-UTR as a target for designing siRNA has been discouraged, because UTR binding proteins and/or translation initiation complexes may interfere with siRNA endonuclease complex binding [19]. There are multiple mutations in cases of PCa being identified throughout the AR coding regions [20-22]. This makes the selection of the AR coding region for siRNA design possibly inappropriate and insufficient to block the expression of mutated AR in PCa patients. We selected three 5'-UTR regions of the AR for our siRNA design; only the region between -25 to -7 bp could successfully suppress AR expression in LNCaP cells (data not shown). Our results further demonstrated that siRNA's efficacy of suppressing gene expression varies from one region to another, due to target sequence structures [23].

### LNCaP cells required AR to support cell proliferation

**Figure 3:**
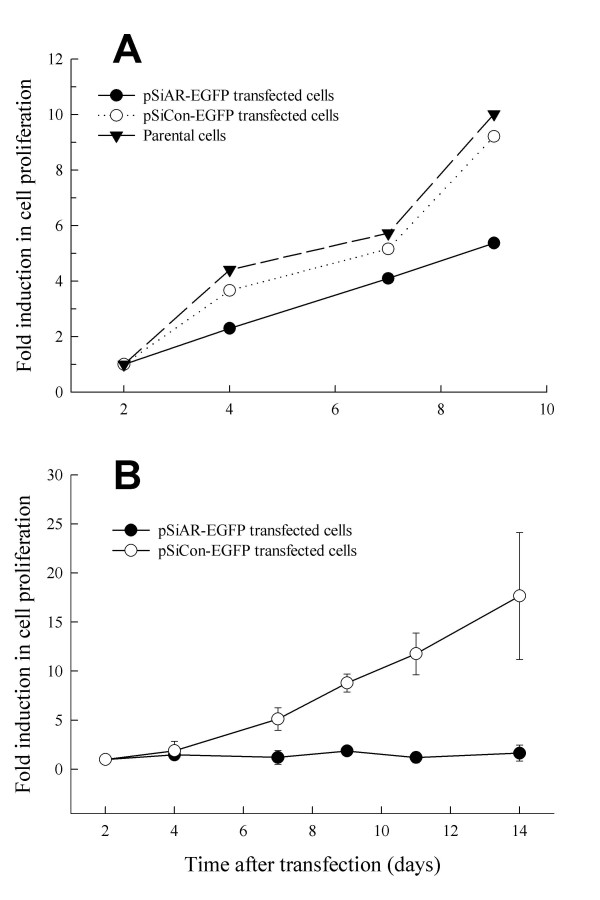
**Suppression of LNCaP cell proliferation in the absence of endogenous AR. **LNCaP cells were seeded in tissue culture plates and transfected with a mixture of either pSiAR-EGFP or pSiCon-EGFP plasmid construct with Lipofecamine 2000 in OPTI-MEM. (A) Cell proliferation without separation of GFP-positive and negative cells. At 24 hours following transfection, cells were trypsinized and distributed into each well (1,000 cells/well) of 96-well tissue culture plates in the presence the complete medium. Cell proliferation was determined using the XTT assay kit for a period of 9 days; data from days 11 and 14 were not included since parental and pSiCon-EGFP transfected LNCaP cells reached confluence after day9. (B) LNCaP cell proliferation following enrichment of GFP-positive cells. At 24 hours after transfection, cells were trypsinized and EGFP-positive cells were collected through the MoStar cell sorting system. GFP-positive cells were seeded into each well (1,000 cells/well) of 96-well plates for cell viability assay. Cell proliferation was determined for a period of 14 days. Data were calculated as absorbance at days following transfection normalized to the absorbance at the day of cell sorting, and presented as fold induction in absorbance. LNCaP cells transfected with pSiCon-EGFP plasmid construct were used as the AR-positive control. * represents significant statistical difference between LNCaP cells with and without AR (*P *< 0.001). Each time point represents the mean ± SEM from 3 independent experiments.

To determine LNCaP cell proliferative capability in the absence of AR, cell proliferation with and without cell sorting was performed following the silencer construct transfection. When cell proliferation was performed without cell sorting, during a 9 day period the pSiAR-EGFP transfected LNCaP cells consistently proliferated slower than the parental cells and the pSiCon-EGFP transfected cells (Figure 3A). After 9 days in culture, both parental and control cells reached confluence. The reduced cell proliferation in pSiAR-EGFP transfected cells might reflect that cells receiving the AR siRNA construct failed to proliferate in the absence of AR.

To confirm that AR signaling is required for LNCaP cell proliferation, GFP enriched LNCaP cells were collected through cell sorting at 24 hours following transfection. LNCaP cells transfected with the pSiCon-EGFP construct continued to grow over the entire experimental period (Figure 3B). This indicates that the silencer control construct did not affect cell proliferation. However, pSiAR-EGFP transfected LNCaP cells (GFP-positive, AR-negative) failed to proliferate (Figure 3B), indicating that the androgen-sensitive prostate cells require AR for proliferation.

Suppression of AR expression and inactivation of AR function in prostate cells has been achieved through the use of an antisense oligonucleotide [12,24], a hammerhead robozyme [13], and a synthesized ds siRNA duplex [18] to target AR mRNA. Microinjection of an AR neutralizing antibody has also been reported to block AR-mediated signaling in LNCaP cells [14]. Recently, the use of vector-based siRNA targeting AR has been reported to study the involvement of AR signaling in vitamin D-suppressed prostate cell growth [25]. These published approaches were intended for transient gene silencing in target cells, and provided short term elimination of AR expression. All of the currently published results are consistent with our observations that disruption of AR signaling adversely affects androgen-sensitive LNCaP cell proliferation.

### LNCaP cells required AR to maintain survival

**Figure 4:**
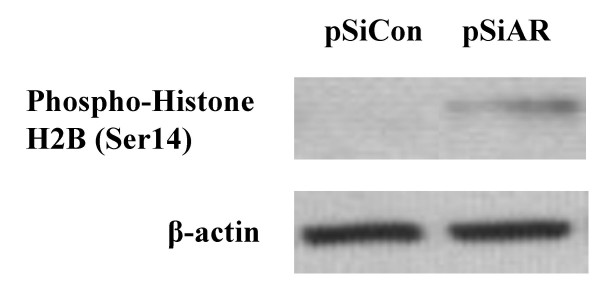
**Elevated expression of phospho-histone H2B S(14) in AR-knockdown LNCaP cells. **LNCaP cells were either transfected with pSiAR-EGFP or pSiCon-EGFP plasmid construct. On day 6 after transfection, cells were harvested. Cellular proteins were extracted using acid extraction method, electrophoresized through gradient Tris-HCl gels, and electroblotted onto PVDF membranes. Levels of phospho-histone H2B expression was detected by an immunoassay procedure.

To determine whether LNCaP cells could survive without AR, we determined the levels of phospho-histone H2B expression and the number of apoptotic body following AR silencing. Histon H2B phosphorylation has been shown to be uniquely associated with apoptosis, specifically in apoptosis-induced chromatin condensation in mammalian cells [26]. Its expression significantly increases in cells undergoing apoptosis [27]. Using Western blot analysis, phospho-histone H2B expression was quantitated in LNCaP cells transfected with either pSiAR-EGFP or pSiCon-EGFP. There was no detectible phospho-histone H2B signal in pSiAR-EGFP or control transfected cells at 4 days after transfection (data not shown). Six days following pSiAR-EGFP transfection, however, elevated levels of phospho-histone H2B expression were detected in AR negative LNCaP cells. At that time, there was no detectible phospho-histone H2B in the pSiCon-EGFP transfected LNCaP cells (Figure 4).

**Figure 5:**
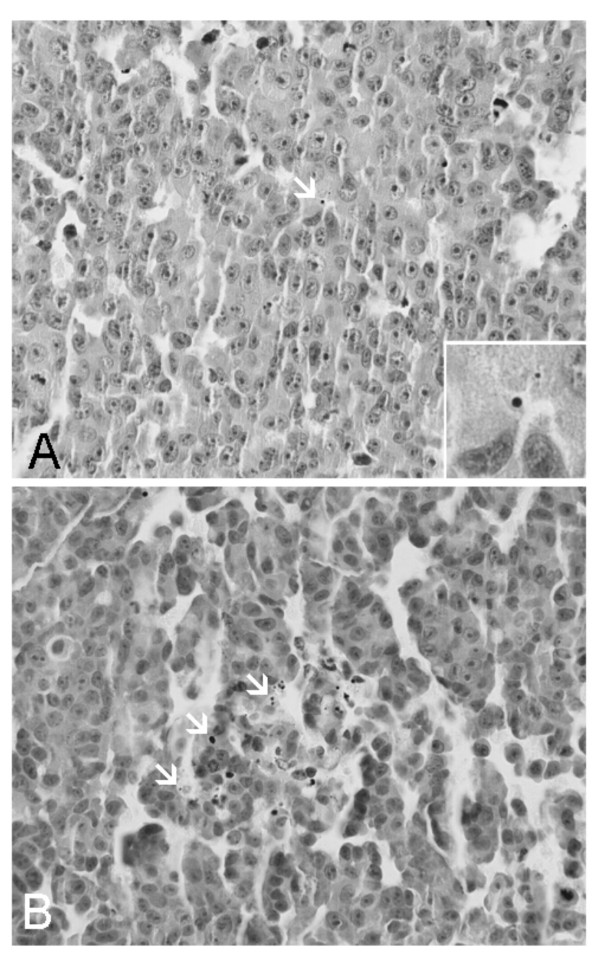
**Elevated number of apoptotic LNCaP cells transfected pSiAR-EGFP construct. **Apoptotic bodies in LNCaP cells transfected with control vector pSiCon-EGFP (A) and pSiAR-EGFP (B). Following transfection with these plasmid constructs, LNCaP cells were collected through centrifugation, fixed in neutralized formalin, and encased in agrose blocks. Cell blocks were paraffin-embedded, sectioned, deparaffinized, rehydrated, and stained with hematoxylin. Apoptotic bodies which showed condensed and/or cleaved nucleus were counted from random fields and numerical data are shown in Table 1.

To prove that AR signaling is required for LNCaP cell survival, the number of cells undergoing apoptosis (demonstrated by the presence of apoptotic bodies) was determined in cells transfected with pSiAR-EGFP and control constructs (Figure 5). The average number of apoptotic bodies in pSiAR-EGFP transfected cells was 52.89 per 1,000 cells, versus 6.53 in 1,000 pSiCon-EGFP transfected cells (Table 1). The transfection efficiencies for each vector were not statistically significant – pSiAR-EGFP was 17.0% and pSiCon-EGFP was 22.9% (Table 1). When the number of apoptotic bodies was corrected for transfection efficiency, ABI was 296.2 for pSiAR-EGFP and 29.2 for pSiCon-EGFP transfected cells (Table 1). The ABI was statistically higher in pSiAR-EGFP transfected cells versus pSiCon-EGFP transfected cells. These results demonstrated that LNCaP cells lacking AR would go through the process of apoptosis.

**Table 1:** Apoptotic body indices in pSiAR-EGFP and pSiCon-EGFP transfected LNCaP cells

	# of apoptotic bodies (per 1,000)^a^	Transfection efficiency (%)	Apoptotic body index^c^
	Mean + S.D.	Mean + S.D.	Mean + S.D.
pSiAR-EGFP	52.89 ± 22.12^b^	17.0 ± 3.45	296.2 ± 78.2^b^
pSiCon-EGFP	6.53 ± 2.85	22.9 ± 2.84	29.2 ± 15.4

^a ^The number of apoptotic bodies were was determined by counting 1,000 cells from each of 3 independent hematoxylin stained slides.

^b ^Significant difference between pSiAR-EGFP and pSiCon-EGFP transfected cells (p < 0.05).

^c ^Apoptotic body indices were calculated after normalizing to transfection efficiency.

To demonstrate that LNCaP cells could survive with transient suppression of AR, we also performed AR protein expression knockdown using a synthesized, pooled ds siRNA, siRNA SMARTpool AR^® ^(Upstate). This siRNA pool suppressed a majority (greater than 95%) of AR protein expression in LNCaP cells at 16 hours after transfection, as compared to cells transfected with a non-specific control pool. The cells maintained suppressed levels of AR protein expression for at least 72 hours following transfection. They resumed AR expression, to 20–30% of normal levels, after 72–96 hours post-transfection (data not shown). LNCaP cells could survive in the absence of AR for at least 3–4 days, as indicated by the lack of detectable phospho-histone H2B signals at 96 hours following the pSiAR-EGFP transfection (data not shown). Due to the transient nature of the use of antisense oligonucleotides, hammerhead robozymes, ds siRNA duplexes, and microinjected neutralizing antibodies against AR, cell survival in the extended absence of AR could not be assessed. Using the combination of the vector-based siRNA delivery and enrichment of AR-negative cells through the selection of GFP-positive cells enabled us to study cell behavior in the absence of AR over an extended time-period.

Antiandrogens are used to prevent the acquisition of a transcriptionally active conformation of the AR. The induction of apoptosis in prostate cells treated with bicalutamide has been observed [28]. However, several reports also indicate that in the presence of bicalutamide, prostate cells survive in culture [29], regain cell growth after an extended period of exposure to the antiandrogen [30,31], and become a more invasive phenotype [32]. Bicalutamide can also act as an AR agonist, resulting in the stimulation of AR *trans*-activation [33]. Furthermore, it has been indicated that bicalutamide may support prostate cell survival and progression through selection of cells with AR mutations [34] or cells with elevated expression of growth factors [35]. These mutations generate receptors that respond to other steroids and antiandrogens by increased *tran*s-activation [4]. These results suggest that antiandrogens may be insufficient to block AR signaling through multiple candidate pathways.

The potential use of RNAi technology has been investigated in the field of gene therapy [36,37]. Targeting AR suppression using RNAi might be more efficient and specific than using antiandrogens to inactivate AR *trans*-activation and turn off ARGs expression. LNCaP cells potentially consist of multiple cell lineages, due to the development of multiple sublines from the original LNCaP cells [38-40]. Although all LNCaP cells transfected by the AR siRNA construct could neither proliferate nor survive in the absence of the AR in this study, we do not have information regarding specific sub-populations that may be more susceptible to transfection and sensitive to the absence of the AR. The development of a more efficient delivery system, such as the lentiviral-based gene delivery system [41], may allow for further study of AR signaling in multiple prostate cell lines.

## Conclusion

We concluded that all PCa cells, at least in the androgen-sensitive and AR-positive stages, require AR for continued proliferation and survival. The identification of AR pathway and ARG(s) that transduce androgen signaling might provide a specific target to block androgen-activated, AR-mediated prostate cell growth. Specifically targeting AR or its downstream signaling molecules potentially will be effective in achieving total androgen blockade. With the development of "long-term" siRNA and efficient delivery systems *in vivo*, we may achieve a total AR blockade in the prostate, thereby improving treatment for patients with prostate diseases.

## Methods

### Establishment of AR silencer plasmid constructs

To establish a siRNA plasmid construct with fluoresce selection marker, a multiple cloning site (MCS) was excised from the pSE380 (Invitrogen) using *Bcl *I and *Hin*d III. The MCS containing fragment was subcloned into *Bam *HI and *Hin*d III linearized pSilencer 2.1-U6 hygro vector (Ambion) to obtain pSilencer 2.1-U6 MCS hygro. The IRES2-EGFP fragment coding for GFP was excised from pIRES2-EGFP (BD Biosciences Clontech) using *Bgl *II and *Afl *II. This excised fragment was then subcloned into the pSilencer 2.1U6 MCS hygro linearized with *Bcl *I and *Sal *I in the presence of a short adaptor containing *Afl *I site on its 5'ends and *Sal *I site on its 3'ends. This established plasmid was named pSilencer 2.1-U6-IRES2-EGFP. To create the silencer construct targeting AR, two complementary strands of oligonucleotides targeting -25 to -7 bp upstream from the ATG transcription start codon of the AR (GenBank accession number M20132; Figure 1A) with *Bgl *II and *Sfi *I sites on its 5' and 3' ends, respectively, was synthesized and annealed. The ds oligonucleotide was cloned into *Bam *HI and *Hin*d III linearized pSilencer 2.1-U6-IRES2-EGFP. The 19-nucleotide AR target sequence is indicated in the sense strand of the synthesized ds DNA. The U6 promoter-driven siRNA expresses the sense and antisense strands of AR siRNA [42] that have a termination signal consisting of 6 thymidines [43].

Since the SV40 promoter could not provide sufficient levels of GFP expression in LNCaP cells for monitoring successful transfection (data not shown), the SV40 promoter was replaced by cymagalovirus (CMV) promoter. The CMV promoter was excised from pIRES2-EGFP with *Nsi *I and *Xho *I, and ligated into *Aat *II and *Xho *I linearized pSilencer 2.1-U6-IRES2-EGFP. The final construct was designated pSiAR-EGFP (Figure 1B). Control construct also was established by cloning an annealed ds DNA fragment with a scrambled 19-nucleotide AR target sequence, named pSiCon-EGFP. The scrambled sequence was subjected to Blast search to ensure no match to any known transcript.

### LNCaP cell culture, transfection, and selection of cells transfected with the AR silencer expression construct

LNCaP cells were purchased from ATCC (CRL-1740). They were cultured and maintained in complete growth medium consisting of RPMI1640 (Invitrogen) supplemented with 10% fetal bovine serum (FBS), 100 unit/ml penicillin, and 100 μg/ml streptomycin (Invitrogen) in a humidified atmosphere containing 5% CO_2 _at 37°C. Transfection of cells with the plasmid silencer constructs was performed in either 60 or 100 mm tissue culture plates using Lipofecamine 2000 (Invitrogen) when cells reached 80–90% confluence. Briefly, for transfection in 100 mm plates, 24 μg of plasmid DNA and 60 μl of Lipofecamine 2000 transfection reagent were diluted separately in 1.5 ml of OPTI-MEM (Invitrogen). They were then combined, and incubated at room temperature for 20 minutes. The mixture was added to LNCaP cells in the presence of 10 ml OPTI-MEM (Invitrogen). The serum free medium was replaced by the complete growth medium 4–6 hours following transfection. The day of transfection was defined as day 0. To select cells that received the silencer constructs, LNCaP cells were trypsinized at 24 hours after transfection (day 1). GFP-positive LNCaP cells were collected using MoStar cell sorting system. For determining the apoptotic index, transfection efficiency was calculated at 24 hours after transfection. A total of 1,000 cells were randomly selected under a microscope equipped with fluorescence filters from 2–3 fields. The percentages of cells expressing GFP were termed "transfection efficiency". The transfection efficiency average was calculated from three independent experiments for each plasmid transfection.

### Immunocytochemical staining of AR in LNCaP cells

To demonstrate that the AR silencer plasmid construct could suppress AR expression in LNCaP cells, at 24 hours post-transfection the cells were re-seeded onto cover slips in 24-well tissue culture plates at the density of 2,000 cells/well. Cells were incubated overnight for adherence. They were sequentially fixed in methanol for 10 minutes and acetone for 1 minute. Cells were then permeabilized with 0.2% triton-X-100 (Simga) in 1x phosphate buffered saline (PBS) at room temperature for 20 minutes. Following incubation with 10% goat serum in 1x PBS to block non-specific binding, cells were incubated for another 1 hour with mouse anti-human AR monoclonal antibody (1:100; NOVOCastra) in 1x PBS containing 10% goat serum. Following three washes with PBS supplemented with 0.5 mM CaCl_2 _and MgCl_2_, cells were incubated with Alexa Fluor^® ^594-conjugated goat anti-mouse IgG (1:200; Molecular Probes) secondary antibody. For nuclei staining, cells were incubated with 0.1 μg/ml 4',6'-diamidino-2-phenylindole hydrochloride (DAPI) at room temperature for 20 minutes. Images were captured by fluorescent microscopy (BX51, Olympus) equipped with the SPOT software (Diagnostic Instrument).

### Protein extraction and Western blot analysis

Western blot analysis was performed to determine AR levels and phospho-histone H2B expression in LNCaP cells transfected with the silencer constructs. To determine levels of AR expression, pSiAR-EGFP and control transfected GFP-positive LNCaP cells were collected through cell sorting. They were lysed with cell lysis buffer consisting of 5 mM EDTA, 0.5% Triton-X-100, and 0.1 mM phenylmethylsulphonylfluoride (PMSF) in 1x PBS at 1 μl/10^4 ^cells. Total cellular extracts were collected following centrifugation. Protein concentrations were determined by bicinchoninic acid (BCA) protein assay kit (Pierce). Aliquots of 20 μg of the protein extracts were separated on 10% Tris-HCl gel (Bio-Rad). Proteins were then transferred to PVDF membranes (Bio-Rad). AR protein was detected by incubating the membranes with the mouse anti-human AR monoclonal antibody (1: 500) at room temperature for 2 hours. This was followed by the horseradish peroxidase (HRP)-conjugated anti-mouse IgG (1:125,000; KPL) secondary antibody incubation at room temperature for another hour. Immunoreactive protein was detected using the enhanced chemiluminescent (ECL) reagent (Pierce) according to the manufacturer's protocol.

To determine histone H2B phosphorylation levels in LNCaP cells in the absence of AR protein, cells were transfected with either pSiAR-EGFP or control construct as described. Cellular proteins were extracted with acid extraction buffer consisting of 10 mM Hepes (pH 7.9), 1.5 mM MgCl_2_, 10 mM KCl, 0.5 mM DTT, and 1.5 mM PMSF in the presence of 0.2 M hydrochloric acid. This was completed according to procedures provided by the antibody supplier (Upstate). Soluble proteins were collected from supernatants after centrifugation at 11,000 × g for 10 minutes. Proteins were then dialyzed overnight against distilled water. Aliquots of 30 μg proteins were prepared from each sample, separated on 4–20% gradient Tris-HCl gels (Bio-Rad), and transferred to PVDF membranes. After blocking non-specific binding, the membranes were incubated overnight with rabbit anti-phospho-histone H2B S(14) antibody (1:500; Upstate) at 4 °C. This was followed by a HRP-conjugated anti-rabbit IgG (1:10,000; Cell Signaling) secondary antibody incubation followed by ECL detection.

### Cell proliferation assay

Cell proliferation assay was determined in pSiAR-EGFP and pSiCon-EGFP transfected LNCaP cells. At 24 hours following transfection, they were either directly seeded into tissue culture plates or subjected to cell sorting to collect GFP-positive cells prior to cell seeding. Cells were seeded into 96-well plates at 1,000 cells/well. Cell proliferation was determined using XTT cell proliferation assay kit (Roche) following the manufacturer's protocol. Proliferation was evaluated for a period of 14 days. Data analysis of three independent transfections of cells sorted for GFP-positive cells was performed as previously reported [44]. Results were presented as mean ± standard error of means (SEM).

### Determination of apoptotic index in cell blocks

To characterize whether LNCaP cells undergo apoptosis in the absence of AR, numbers of apoptotic bodies were quantitated in LNCaP cells transfected with pSiAR-EGFP and control silencer constructs. Transfected cells were tripsinized and centrifuged at 400 × *g *for 5 minutes to collect the cells. The cell pellets were fixed in 10% formalin for at least 24 hours. They were then mixed with drops of warm 2% agarose in order to form cell-agarose blocks. The cell blocks were paraffin embedded, cut at 4–6 μm, mounted on microscope slides, and baked at 55 °C overnight. The slides were deparaffinized, rehydrated to water, and stained by hematoxylin. Apoptotic bodies were quantitated under a bright field microscope at 400× magnification. Apoptotic bodies were defined as small, roughly spherical or ovoid cytoplasmic fragments, some of which contained nuclear fragments [45]. The density of apoptotic cells was determined by counting 1,000 cells from each transfection. Quantitative analyses of apoptotic changes were recorded as apoptotic body indices (ABI, the number of apoptotic cells/transfection efficiencies).

### Statistical Analysis

For statistical analysis, data were presented as mean and SEM from at least 3 independent experiments. Statistical significance was determined when P < 0.05. Student t-test was used for comparing two treatment groups.

## Abbreviations

5α-DHT: 5α-dihydrotestosterone; AR: androgen receptor; ARE: androgen response element; ARG: androgen response gene; RNAi: RNA interference; siRNA: small interfering RNA.
